# VDR regulates simulated microgravity-induced atrophy in C2C12 myotubes

**DOI:** 10.1038/s41598-022-05354-0

**Published:** 2022-01-26

**Authors:** Ryo Yuzawa, Hiroyuki Koike, Ichiro Manabe, Yumiko Oishi

**Affiliations:** 1 Department of Biochemistry & Molecular Biology, Nippon Medical School, 1-1-5 Sendagi, Bunkyo-ku, Tokyo 113-8602 Japan; 2 Department of Systems Medicine, Chiba University Graduate School of Medicine, 1-8-1 Inohana, Chuo-ku, Chiba, 260-8670 Japan

**Keywords:** Medical research, Experimental models of disease

## Abstract

Muscle wasting is a major problem leading to reduced quality of life and higher risks of mortality and various diseases. Muscle atrophy is caused by multiple conditions in which protein degradation exceeds its synthesis, including disuse, malnutrition, and microgravity. While Vitamin D receptor (VDR) is well known to regulate calcium and phosphate metabolism to maintain bone, recent studies have shown that VDR also plays roles in skeletal muscle development and homeostasis. Moreover, its expression is upregulated in muscle undergoing atrophy as well as after muscle injury. Here we show that VDR regulates simulated microgravity-induced atrophy in C2C12 myotubes in vitro. After 8 h of microgravity simulated using 3D-clinorotation, the VDR-binding motif was associated with chromatin regions closed by the simulated microgravity and enhancer regions inactivated by it, which suggests VDR mediates repression of enhancers. In addition, VDR was induced and translocated into the nuclei in response to simulated microgravity. VDR-deficient C2C12 myotubes showed resistance to simulated microgravity-induced atrophy and reduced induction of FBXO32, an atrophy-associated ubiquitin ligase. These results demonstrate that VDR contributes to the regulation of simulated microgravity-induced atrophy at least in part by controlling expression of atrophy-related genes.

## Introduction

Dysfunction within bone, muscle, and the immune system are consequences often related to aging. Loss of skeletal muscle mass results in weakness, reduced mobility and impaired quality of life. Indeed, long‐term muscle wasting is a major risk factor linked to increased mortality^1^. Muscle mass is regulated by the balance between protein synthesis and degradation. In situations such as diabetes, fasting, cancer-bearing, denervation and disuse, protein degradation exceeds protein synthesis, causing muscle atrophy. Of note, the fact that extended spaceflight or lack of physical activity on the ground, both of which are characterized by reduced mechanical loading, cause muscle atrophy indicates that resisting gravity (mechanical force) is indispensable to maintaining healthy muscle.

The two major proteolytic systems, the ubiquitin–proteasome and autophagy-lysosome systems, have been shown to mediate muscle atrophy^2^. The ubiquitin–proteasome pathway is weakly active in the normal physiological state^3^. During muscle atrophy, however, the cardiac and skeletal muscle-specific E3 ubiquitin ligases FBXO32 (Atrogin-1) and TRIM63 (MuRF1) are induced and are responsible for the ubiquitination and proteasomally mediated degradation of myofilaments^3–5^. Mice lacking either *Trim63* or *Fbxo32* show resistance to muscle atrophy induced by denervation, confirming these enzymes’ essential contribution to the pathogenesis of muscle atrophy^4,6^. However, activation of the ubiquitin–proteasome system appears to vary depending on the stimulus. For instance, deletion of *Trim63* in mice does not prevent muscle atrophy during spaceflight. This suggests the mechanism for the muscle atrophy that occurs under microgravity differs from that observed in ground-based models^7^.

Vitamin D receptor (VDR) is a ubiquitously expressed nuclear receptor that is activated upon binding active vitamin D (e.g., 1, 25-dihydroxyvitamin D3) as a ligand. While VDR is well known to regulate calcium (Ca^2+^) and phosphate (Pi) homeostasis to maintain bone, recent studies have shown that it also directly affects skeletal muscle. For instance, overexpression of VDR induces skeletal muscle hypertrophy^8^, whereas systemic^9,10^ or skeletal muscle-specific^11,12^ deletion of *Vdr* reduces muscle fiber size and strength in mice. Moreover, lentivirus-mediated VDR knockdown in mature skeletal muscle induces muscle atrophy in rats, presumably through activation of autophagy, while the major atrophy-related ubiquitin ligases are either downregulated or unchanged^13^. These findings suggest VDR is involved in skeletal muscle development and homeostasis. On the other hand, VDR protein is reportedly upregulated by denervation-induced atrophy^14^. In addition, VDR levels are significantly increased following resistance exercise that causes minor damage to myotubes^15^. Although these results suggest VDR may also have a role in the muscle atrophy and/or damage response, this possibility has not been formally tested. Importantly, nuclear receptors may act differently depending on whether or not their ligands are bound. Indeed, VDR reportedly acts as a repressor in its unliganded state^16^. Moreover, even liganded VDR has been shown to transrepress certain target genes^15^. However, the potential role played by repressive VDR has not been addressed in skeletal muscle. Collectively, the findings summarized above suggest VDR may have dual roles in skeletal muscle physiology and pathology.

In the present study, we explored the transcriptional regulatory mechanisms underlying microgravity-induced muscle atrophy. We found that VDR is induced by simulated microgravity and translocated to the nuclei without addition of exogenous ligand, that VDR binding elements are associated with enhancers that are inactivated by simulated microgravity, and that VDR-deficient C2C12 myotubes established using the CRISPR/Cas9 system are resistant to simulated microgravity-induced atrophy. These findings indicate that VDR regulates simulated microgravity-induced atrophy in vitro.

## Results

### VDR binding motifs are enriched in chromatin regions closed by simulated microgravity

**Figure 1:**
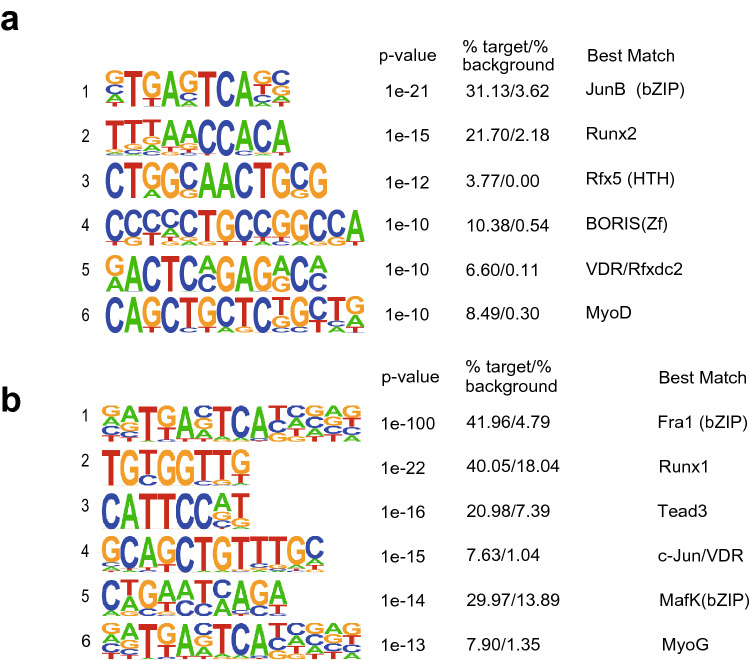
VDR motif is enriched in gene regions closed by simulated microgravity. (**a**) De novo motifs in the chromatin regions identified as regions closed by simulated microgravity in C2C12 myotubes. In the sequence logos, the height of each letter represents the relative frequency of the occurrence of the nucleotide at that position. (**b**) De novo motifs of the chromatin regions identified by ATAC-seq as being simulated microgravity-closed regions and as having significantly reduced H3K27Ac levels. Frequently occurring bases are represented by large letters, while infrequently occurring bases are represented by small letters.

To start identifying epigenetic regulatory mechanisms mediating microgravity-induced atrophy, we cultured, differentiated and fused C2C12 myotubes in a 3D-clinostat apparatus, which creates a simulated microgravity (10^−3^ *g*) environment and, in turn, induces atrophy in vitro^17^. After 48 h of simulated microgravity, C2C12 myotubes showed an atrophied phenotype characterized by longitudinal shortening. This phenotypic change was accompanied by elevated transcription of *Fbxo32* encoding an E3 ubiquitin ligase, one of the key ubiquitin ligases involved in muscle atrophy^18^. To identify transcription factors controlling the programs regulating the simulated microgravity-responsive genes, we performed accessible chromatin-sequencing (ATAC-seq) with C2C12 myotubes cultured for 8 h under clinorotation. In total, 25,961 peaks were identified through ATAC-seq. De novo motif analysis showed that ATAC-seq peaks corresponding to regions closed in response to the simulated microgravity (112 peaks) were most highly enriched for the 5353 motifs of JunB, followed by Runx2. Interestingly, the binding motif corresponding to VDR was ranked 5th (Fig. 1a).

We next performed chromatin immunoprecipitation sequencing (ChIP-seq) for the acetylation of histone H3 lysine 27 (H3K27ac), a dynamic histone modification that correlates with enhancer activity^19^, in C2C12 myotubes cultured for 24 h under simulated microgravity or control conditions. We then analyzed the motifs enriched in the ATAC-seq peaks and the surrounding H3K27ac levels under simulated microgravity. These motifs likely contain the transcription factor binding regions within the simulated microgravity-inactivated enhancers. To focus on enhancers, we eliminated the ATAC peaks in the vicinity of transcription start and termination sites. Of the remaining 16,085 ATAC peaks, 405 showed decreased levels of H3K27ac under simulated microgravity. We also found that the VDR binding motif was ranked 4th among the motifs identified as ATAC peaks inactivated by simulated microgravity (Fig. 1b). These observations suggest that VDR may mediate transcriptional repression of genes in response to simulated microgravity.

### VDR is upregulated in C2C12 myotubes under microgravity

**Figure 2:**
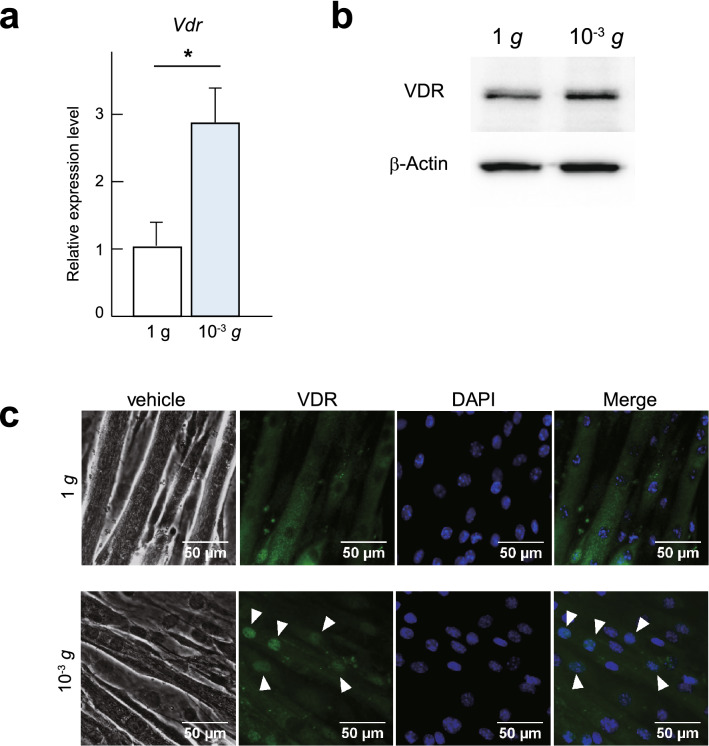
VDR is induced in response to simulated microgravity. (**a**) *Vdr* mRNA expression in C2C12 myotubes cultured for 48 h at 1 *g* or under simulated microgravity (10^−3^ *g*). Data are means ± s.d. n = 5. Mann–Whitney test. *P < 0.05. (**b**) Western blot of VDR and β-Actin in C2C12 myotubes cultured for 48 h at 1 *g* or under simulated microgravity (10^−3^ *g*). Representative images of at least 3 independent experiments are shown. (**c**) Phase contrast images and immunocytochemical staining for VDR (green) in C2C12 myotubes cultured for 48 h at 1 *g* or under microgravity (10^−3^ *g*). Nuclei were counterstained with DAPI (blue). Scale bars, 50 μm.

The observation that VDR binding regions respond to simulated microgravity prompted us to investigate the role of VDR in more detail. We first investigated the effect of simulated microgravity on expression of VDR. We found that under simulated microgravity, VDR expression in C2C12 myotubes is upregulated at the mRNA level and mildly upregulated at the protein level (Fig. 2a,b). These results are consistent with an earlier report showing that the expression level of VDR protein is increased after denervation-induced muscle atrophy^14^.

VDR is a nuclear receptor localized in the cytosol in its unliganded form but is translocated into the nucleus upon ligand binding^20^. However, it has also been reported that VDR is present in the nucleus in the unliganded state and that the unliganded receptor functions as a transrepressor under some conditions. For example, it inhibits cell proliferation during the early stage of myotube differentiation and represses several VDR target genes involved in calcium homeostasis^16,20,21^. With that as context, we performed immunostaining to assess the subcellular localization of VDR. Our immunohistochemistry results showed that VDR is primarily localized in the cytosol in differentiated myotubes maintained in a normal steady state (1 *g*). Interestingly, although we did not add active Vitamin D as a ligand or replace the culture medium, following 48 h of culture under simulated microgravity, VDR was mainly localized in the nuclei (Fig. 2c). Thus, VDR is upregulated and translocated into the nucleus under simulated microgravity without addition of ligand.

### *Vdr* deletion does not affect C2C12 myotube differentiation

**Figure 3:**
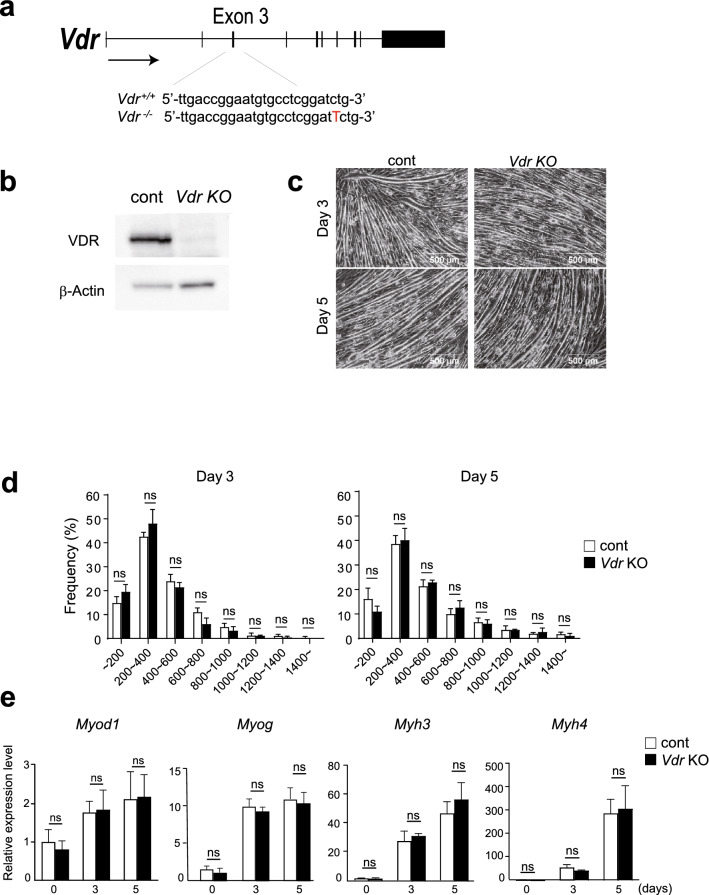
VDR deficiency does not affect myotube differentiation. (**a**) Diagram showing *Vdr* deletion using the CRISPR-Cas9 system. (**b**) Western blot of VDR and β-Actin in C2C12 myotubes confirming that VDR expression was absent in *Vdr* KO cells. (**c**) Phase contrast images of control and *Vdr* KO C2C12 cells after differentiation for 3 or 5 days. Scale bars; 500 μm. (**d**) Distribution of myotube lengths. Data are means ± s.d. Mean numbers of myotubes within the indicated length ranges per 100 myotubes in control and *Vdr* KO C2C12 cells after differentiation for 3 or 5 days. Data are means ± s.d. Two-way ANOVA and multiple Mann–Whitney tests for multiple comparisons. ns: no significant. (**e**) *Myod1, Myog, Myh3 and Myh4* mRNA expression in control and *Vdr* KO C2C12 cells after differentiation for 0, 3, or 5 days. Data are means ± s.d. Two-way ANOVA and multiple Mann–Whitney tests for multiple comparisons. ns: not significant.

To investigate the function of VDR, we used CRISPR/Cas9 technology to generate *Vdr* knockout (KO) C2C12 cell lines. One of those had a single nucleotide insertion within exon 3 of the *Vdr* gene without additional mutations at predicted off-target sites (Fig. 3a). Western blotting showed that VDR protein expression was completely abrogated in the KO cells (Fig. 3b).

Before using *Vdr* KO C2C12 myotubes for analyses related to atrophy, we first tested whether *Vdr* deletion affects myotube formation, as previous studies suggested a role for VDR in muscle development in vivo and myogenic differentiation in vitro^22–25^, though there is a conflicting report^13,26^. *Vdr* KO and control C2C12 cells were cultured in differentiation medium for 3 or 5 days. As shown in Fig. 3c,d, we detected no differences in the morphology or length of myotubes between *Vdr* KO and control cells. Similarly, mRNA expression levels of the myogenic regulatory factor genes *Myod1* and *Myog* as well as the myotube markers *Myh3* and *Myh4* were comparable on days 3 and 5 of differentiation (Fig. 3e). These results demonstrate that *Vdr* deletion does not affect myotube differentiation in C2C12 cells.

### *Vdr* deletion protects against simulated microgravity-induced atrophy

**Figure 4:**
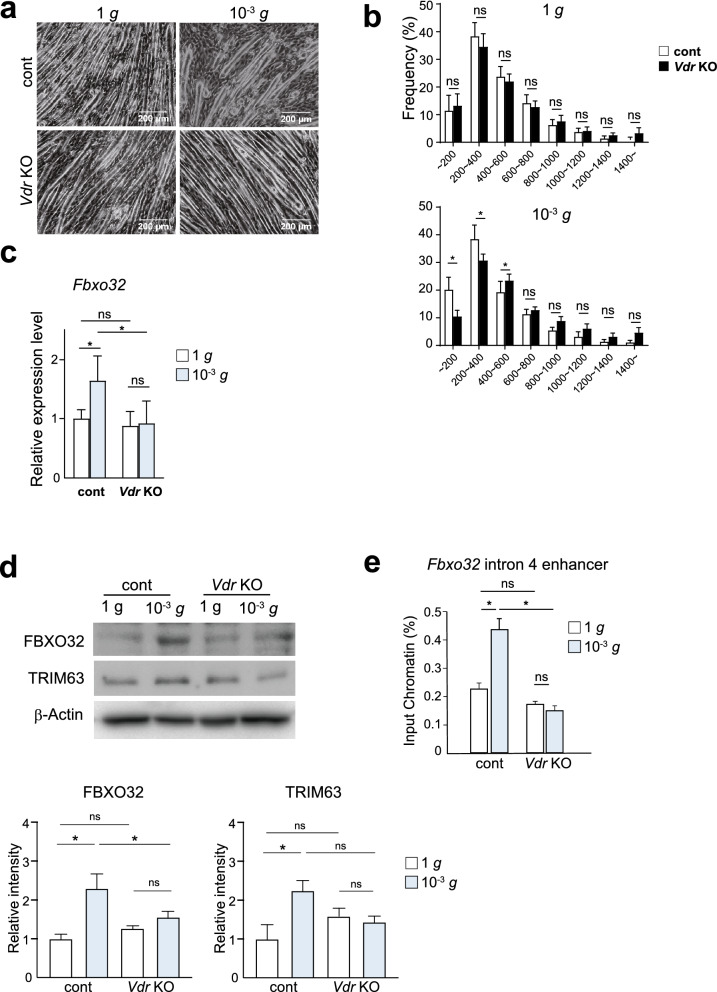
VDR-deficient myotubes are resistant from simulated microgravity-induced atrophy. (**a**) Phase contrast images of control and *Vdr* KO C2C12 myotubes cultured for 48 h under control (1 *g*) or simulated microgravity (10^−3^ *g*) conditions. The culture conditions were identical between the two groups, except for the use of the 3D-clinostat apparatus. Scale bars: 200 μm. (**b**) Distribution of myotube lengths. Data are means ± s.d. Mean numbers of myotubes within the indicated length ranges per 100 myotubes in control and *Vdr* KO C2C12 cells at 1 *g* (control) or under simulated microgravity (10^−3^ *g*). Data are means ± s.d. Two-way ANOVA and multiple Mann–Whitney test for multiple comparisons. *P < 0.05. (**c**) *Fbxo32* mRNA expression in control and *Vdr* KO C2C12 myotubes cultured for 48 h at 1 *g* or under simulated microgravity (10^−3^ *g*). Data are means ± s.d. Two-way ANOVA for multiple comparisons. *P < 0.05. (**d**) Western blot of FBXO32, TRIM63 and β-Actin in control and *Vdr* KO C2C12 myotubes cultured for 48 h at 1 *g* or under simulated microgravity (10^−3^ *g*). Images representative of at least 3 independent experiments are shown. Relative band intensities corresponding to FBXO32 and TRIM63 are shown in the bar graph, n = 3. One-way ANOVA and Tukey’s test for multiple comparisons. *P < 0.05, ns: not significant. (**e**) ChIP-qPCR analysis of the association of H3K27Ac with the Fbxo32 intron 4 enhancer in control and *Vdr* KO C2C12 myotubes cultured for 24 h at 1 *g* or under simulated microgravity (10^−3^ *g*). Data are means ± s.d. one-way ANOVA and Tukey’s test for multiple comparisons. *P < 0.05. ns: not significant.

We then assessed the effects of VDR-deficiency on simulated microgravity-induced atrophy. Differentiated and fused *Vdr* KO and control C2C12 myotubes were exposed to simulated microgravity for 48 h. In control myotubes, simulated microgravity increased shorter myotube fractions; in particular, the fractions of myotubes ≤ 400 μm in length were significantly increased (Fig. 4a,b), as were levels of both FBXO32 mRNA and protein (Fig. 4c,d). In sharp contrast, no myotube shortening or induction of FBXO32 was observed in *Vdr* KO myotubes (Fig. 4a–d). Levels of TRIM63 protein were moderately elevated in response to simulated microgravity in the control cells but not in *Vdr* KO cells. Collectively, these results show that *Vdr* deletion from C2C12 myotubes inhibits simulated microgravity-induced atrophy.

To gain insight into the molecular mechanisms underlying the differential *Fbxo32* expression in the two genotypes, we performed ChIP-PCR to look at H3K27ac^19^. The basal levels of H3K27ac at the *Fbxo32* intron 4 enhancer were comparable in control and *Vdr* KO cells. By contrast, H3K27ac was clearly increased in control myotubes after 24 h of simulated microgravity but was unchanged in *Vdr* KO myotubes (Fig. 4e). It thus appears the reactivity of the *Fbxo32* enhancer to microgravity is lost from VDR-deficient cells. These findings suggest that in the absence of exogenous active vitamin D (1,25(OH)2D3) as a ligand, VDR plays an important role sensing external stresses that may cause muscular atrophy of C2C12 myotubes.

## Discussion

In the present study we showed that VDR is involved in simulated microgravity-induced myotube atrophy in vitro. VDR was upregulated by simulated microgravity and translocated to the nuclei (Fig. 2a–c), suggesting it responds to a simulated change in gravity. ATAC-seq and ChIP-seq experiments showed that VDR binding elements associate with enhancers that were inactivated by simulated microgravity (Fig. 1a,b). Moreover, VDR deficiency suppressed simulated microgravity-induced atrophy (Fig. 4a–d). Collectively, these results demonstrate that VDR contributes to muscle atrophy induced by simulated microgravity, at least in part by regulating expression of atrophy-related genes.

Our finding that VDR binding motifs were enriched in enhancers inactivated by simulated microgravity suggests VDR mediates repression of gene transcription during muscle atrophy. Although previous studies have mainly focused on the transactivating function of liganded VDR, ligand-bound VDR also reportedly acts as a transrepressor^27,28^. In addition, unliganded VDR may negatively regulate target gene transcription by recruiting SMRT corepressor^21,29^. Because we did not add active vitamin D as a ligand to the culture medium, it is possible that unliganded VDR acted as a repressor in atrophying myotubes. Future studies will be needed to directly test whether unliganded VDR mediates the muscle atrophy program.

Notably, simulated microgravity promoted nuclear translocation of VDR in myotubes (Fig. 2c). Upon ligand binding, VDR forms a heterodimeric complex with the retinoic acid receptor (RXR), which facilitates nuclear translocation and subsequent binding of the complex to specific vitamin D response elements^30–33^. In our model, VDR translocation was promoted by simulated microgravity without addition of exogenous vitamin D. This is consistent with an earlier report that in several cell types, including MCF-7 breast cancer cells, VDR is localized within the nuclei in the absence of ligand^16^. Moreover, our ATAC-seq results show that the VDR binding motif is enriched in chromatin regions closed by simulated microgravity and suggest involvement of nuclear VDR (Fig. 1a,b). It will be important to further elucidate the mechanism by which VDR translocation is regulated by microgravity independently of VDR ligands. It will also be important to determine whether VDR translocation occurs in other models of muscle atrophy, such as the atrophy associated with disuse, denervation, and metabolic alterations.

In the present study, we used cells subjected to microgravity simulated using a 3D-clinostat instrument, not spaceflown cells. The 3D-clinostat has been widely used to simulate microgravity at ground level; however, the rotation also causes fluid motion within the culture flask, which can potentially impose shear stress on the cells^34^. Although only a small portion of the cells were exposed to levels of shear stress calculated to be sufficient to affect endothelial or smooth muscle cells, the possible involvement of mechanical stress cannot be immediately excluded. Nonetheless, we think it is noteworthy that the use of 3D-clinorotation has enabled us to analyze possible roles of VDR in muscle atrophy due not only to microgravity but also to disuse. This method was successfully used to analyze the molecular mechanisms of the disuse responses in both bone and skeletal muscle cells^18^. To further elucidate the biological functions of VDR in muscle atrophy, future studies will need to assess the role of VDR in in vivo models, such as the hind-limb suspension model.

## Materials and methods

### Antibodies

For western blotting, anti-VDR (D-6): sc-13133, anti-Fbxo32/MAFbx (F-9): sc-166806, anti-Trim63/MuRF1 (C-11): sc-398608 (Santa Cruz), and anti-β-Actin: 010-27841 (Wako) antibodies were used.

### Cell culture

C2C12 mouse skeletal muscle cells (ATCC #CRL-1772, passage numbers 6–8, Rockville, MD) were cultured at 37 °C under 5% CO_2_ in Dulbecco’s modified Eagle medium (DMEM) supplemented with 10% fetal bovine serum (GE Healthcare) and 1% penicillin–streptomycin (Nacalai Tesque). Cells were passaged every 3 days. To induce differentiation, cells were plated at a density of 3.0 × 10^4^ cells/cm^2^ and incubated for 1 day in growth medium (DMEM, 10% FBS). Then after confirming that the cells had reached 90% confluence, the medium was switched into differentiation medium consisting of DMEM supplemented with 2% horse serum (GE Healthcare) and 1% penicillin–streptomycin. The cells were incubated in the differentiation medium for 3 days to induce myotube formation and fusion, after which they were used for experimentation.

### 3D-Clinorotation culture

Simulated microgravity conditions were produced using a 3D-clinorotation apparatus (Gravite^®^, Space Bio-Laboratories Co., Ltd.) as descried previously^35,36^. This device produces an environment similar to that of outer space (10^−3^ *g*) by rotating a sample around two axes, integrating a gravity vector with a temporal axis. This is accomplished by rotation of a chamber at the center of the device, which results in uniform dispersion of the gravity vector within a spherical volume, at a constant angular velocity. Within 8 min, these conditions produced a simulated environment of 10^−3^ *g* when measured with a gravity acceleration sensor and were defined as simulated microgravity. Differentiated C2C12 myotubes were cultured in a cell culture flask (T-75, Corning) filled with differentiation medium for 8, 24, or 48 h under simulated microgravity at 37 °C. C2C12 myotubes cultured in the same cell culture flask under normal ground conditions served as a control.

### ATAC-seq

Approximately 50,000 cells were washed once with PBS and once with cold lysis buffer (10 mM Tris–HCl (pH 7.4), 10 mM NaCl, 3 mM MgCl_2_, 0.1% IGEPAL CA-630). The cells were then suspended in 50 μl of 1x reaction buffer (25 μl of Tagment DNA Buffer, 2.5 μl of Tagment DNA enzyme I, and 22.5 μl of water) (Nextera DNA Library Preparation Kit, Illumina) as previously described^37^. Transposase reactions were carried out for 30 min at 37 °C, and the DNA was immediately purified using a PCR purification Kit (Qiagen). The DNA was amplified for 9–11 cycles using Nextera primer Ad1 and a unique Ad2.n barcoding primer with NEBNext High-Fidelity 2XPCR Master Mix (NEB). The resulting libraries were size selected and then single end sequenced using a HiSeq 1500 sequencer (Illumina) for 51 cycles according to the manufacturer’s instructions.

Reads were aligned with the mm9 mouse genome using STAR^38^. Peak detection and de novo motif analysis were carried out using HOMER^39^. Peaks that overlapped blacklisted regions^40^ or simple repeat regions were removed. For identification of ATAC peaks affected by microgravity, peaks detected in samples incubated under 1 g and under microgravity were merged. Counts within 400 bp around the merged ATAC peaks were calculated. The ATAC peaks with counts in the simulated microgravity sample that were more than 2-fold greater than those in the control samples were identified using Homer’s getDifferentialPeaks function and used as simulated microgravity-opened peaks that were subjected to de novo motif analysis.

### ChIP-seq

Ten million C2C12 cells were used for ChIP-seq. Briefly, the cells were crosslinked in 1% formaldehyde, after which they were collected and resuspended for 5 min in swelling buffer (10 mM HEPES/KOH pH 7.9, 85 mM KCl, 1 mM EDTA, 0.5% IGEPAL CA-630) containing protease inhibitors. The cells were then spun down and resuspended in 500 µl of lysis buffer (50 mM Tris/HCL pH7.4, 1% SDS, 0.5% Empigen BB, 10 mM DETA) containing protease inhibitors, and the chromatin was sheared by sonication. The lysate was diluted with 750 µl of dilution buffer (20 mM Tris/HCl, 100 mM NaCl, 0.5% TritonX-100, 2 mM EDTA), after which 1% was taken as input DNA, and immunoprecipitation was carried out overnight using Dynabeads protein G (Thermo Fisher) coated with specific antibody. Thereafter, the beads were washed two times each with wash buffer I (20 mM Tris/HCl, 150 mM NaCl, 0.1% SDS, 1% Triton X-100, 2 mM EDTA), wash buffer II (10 mM Tris/HCl, 250 mM LiCl, 1% IGEPAL CA-630, 0.7% Na-deoxycholate, 1 mM EDTA), TE plus 0.2% triton X-100, and TE plus 50 mM NaCl, after which the beads were eluted with elution buffer containing 1% SDS. DNA was reverse crosslinked and purified using a QIAquick PCR purification Kit (Qiagen) according to the manufacturer's instructions.

To focus on potential enhancers, ATAC peaks in the vicinity of the transcription start and stop sites were removed. Using the remaining 16,085 peaks we analyzed differential depositions of H3K27ac reads within 600 bp of the ATAC peaks using Homer’s getDifferentialPeaks function. The ATAC peaks that had > 2-fold changes in H3K27ac levels were identified.

### Generation of *Vdr*-KO C2C12 cells

A *Vdr*-KO C2C12 cell clone was made using a Clustered Regularly Interspaced Short Palindromic Repeats /CRISPR-Associated Proteins 9 (CRISPR-Cas9) system^41^. The guide RNA was designed to target exon 3 of the mouse *Vdr* genome as follows:

gRNA (Fwd): 5′-CACCGCGGAATGTGCCTCGGATCTG-3′

gRNA (Rev): 5′-CGCCTTACACGGAGCCTAGACcaaa-3′

The pair of oligonucleotides were ligated into the pSpCas9(BB)-2A-GFP plasmid (Origene PX458), which was then purified with Midi Plus^™^ Ultrapure Plasmid Extraction System (Viogene). Lipofectamine 2000 (Invitrogen: 11668-019) was then used according to the manufacturer's instructions to transfect undifferentiated C2C12 cells with the plasmids carrying gRNA. In parallel, undifferentiated C2C12 myoblasts transfected with non-silencing, control plasmid were generated as a control. Two days after transfection, the cells expressing GFP were selected through fluorescence-activated cell sorting (FACS Aria II cell sorter; BD Bioscience). Sorting of GFP-positive cells was independently performed at least three times. The cells obtained were cultured separately, after which insertion of a single nucleotide within exon 3 of *Vdr*-KO cells was confirmed by DNA sequencing.

### Real-time PCR analysis

Total RNA was isolated from cultured cells using ISOGEN (NIPPON GENE) according to the manufacturer’s instructions. cDNA was synthesized using ReverTra Ace qPCR RT Master Mix with gDNA Remover (TOYOBO). All qPCR protocols were performed with StepOne Plus (Applied Biosystems) using a KAPA SYBR FAST ABI Prism qPCR Kit (Kapa Biosystems). Primers are listed in Supplemental Table 1. Expression of *Actb* was used as an internal control.

### Measurements of myotube length

Images of myotube cells (magnification: 40x or 100x) were obtained using an inverted microscope (OLYMPUS: IX73) and imaging software for microscopes (OLYMPUS: CellSens Standard 1.9). The lengths of those myotubes were quantified using ImageJ software (version 1.52a, NIH, USA). Four or more fields were chosen randomly, and more than 120 myotubes were measured and aggregated^42–46^.

### Immunocytochemistry

For immunostaining of VDR, myotubular cells were washed twice with 1x PBS and fixed for 15 min in 4% paraformaldehyde (PFA). After washing twice with 1x PBS, the cells were treated for 5 min with 0.1% of Triton X-100 (Sigma-Aldrich: T8787-250ML) in PBS to permeabilize the membranes, then washed again twice with 1x PBS and incubated for 1 h with 2% BSA (Wako 013-27054) to block nonspecific reactivity. Next, the cells were immersed in anti-VDR antibody (Santa Cruz Biotechnology: sc-13133) diluent, and the antigen–antibody reaction was allowed to proceed overnight at 4 °C. After washing the cells three times with 1x PBS, fluorescent antibody (Alexa Flour 488, Invitrogen: A-11017) diluent was added allowed to react for 1 h at room temperature under light shielding. Finally, the cells were washed three times with 1x PBS and counterstained with 0.2 µg/ml DAPI diluent for 10 min at room temperature under light shielding. Images were taken with an inverted fluorescence microscope (OLYMPUS: IX73) and imaging software for microscopes (OLYMPUS: CellSens Standard 1.9).

### Western blot

To prepare whole lysates for Western blotting, wild-type or *Vdr*-KO C2C12 cells in culture flasks were washed twice for 5 min each with 2 mL of ice-cold 1x PBS, after which they were lysed in 150 µl of RIPA SDS buffer (150 mmol/l NaCl, 50 mmol/l HEPES, 1% NP-40, 0.5% sodium deoxycholate, 0.1% SDS, 5 mmol/l EDTA (pH 8.0), 1% protease inhibitor Cocktail (Roche) and PMSF). The cells were then treated for 5 cycles in which each cycle consisted of 30 s of sonication and 30 s of cooling on ice. The resultant cell suspensions were centrifuged at 15,000 rpm, 4 °C, 10 min, and their supernatants were collected as whole lysates. The protein concentrations in the lysates were measured using a BCA assay kit (Thermo Scientific, 23227), after which the lysates were diluted to 30 ng of protein/20 µl with RIPA SDS buffer and heated in 6x Sample Buffer for 3 min at 98 °C for denaturation. The denatured lysates were subjected to electrophoresis in 10% SDS-PAGE gel and transferred to PVDF membranes (120 mA for 1 h). After blocking, the membranes were incubated first with diluted primary antibody overnight at 4 °C with rotation and then with HRP-linked anti-mouse secondary antibody (1:10,000 dilution; GE Healthcare) for 1 h. Immunoreactive bands were detected with ECL™ Prime Western Blotting Detection Reagent (GE Healthcare) using a FUSION SL4 imaging system (Vilber Lourmat).

### Statistics and reproducibility

Statistical analyses were performed using Graph Pad Prism 9 software. Images were prepared using Adobe Illustrator CS5 and Photoshop CS5.1. Sample sizes were not based on power calculations. Data are presented as the mean ± s.d, except where otherwise indicated. For experiments involving two factors, data were analyzed using two-way ANOVA and multiple Mann–Whitney tests, except where otherwise indicated. Individual pair-wise comparisons were made using the Mann–Whitney t test. Values of *P* < 0.05 were considered significant.

## Supplementary Information

Supplementary Information.

## Data Availability

All ATAC-seq and ChIP data are available in the GEO under accession numbers GSE184907.
