# Supplementary material for: VDR regulates simulated microgravity-induced atrophy in C2C12 myotubes
Source: Sci Rep. 2022 Jan 26;12:1377. doi: 10.1038/s41598-022-05354-0 (PMC8791983; doi:10.1038/s41598-022-05354-0)
Supplement: Supplementary file 1 — Supplementary Information. [file 41598_2022_5354_MOESM1_ESM.pdf]

Supplementary Table

qPCR primers used in the study

	Forward	Reverse
qPCR		
Actb	GGCTGTATTCCCCTCCATCG	CCAGTTGGTAACAATGCCATGT
Fbxo32	GAGTGGCATCGCCCAAAAGA	TCTGGAGAAGTTCCCGTATAAGT
Trim63	GTGTGAGGTGCCTACTTGCTC	GCTCAGTCTTCTGTCCTTGGA
Myog	AGTGAATGCAACTCCCACAG	ACGATGGACGTAAGGGAGTG
MyoD	GGCTACGACACCGCCTACTA	GAGATGCGCTCCACTATGCT
Myh3	AAAAGGCCATCACTGACGC	CAGCTCTCTGATCCGTGTCTC
Myh4	GCTTGAAAACGAGGTGGAAA	CCTCCTCAGCCTGTCTCTTG
Fbxo32 intron 4	CCTCATGGACTGAAGAGCGG	GGACAGAGTGACCTGGCAAA

The sequence of qPCR primers used in this study is listed.

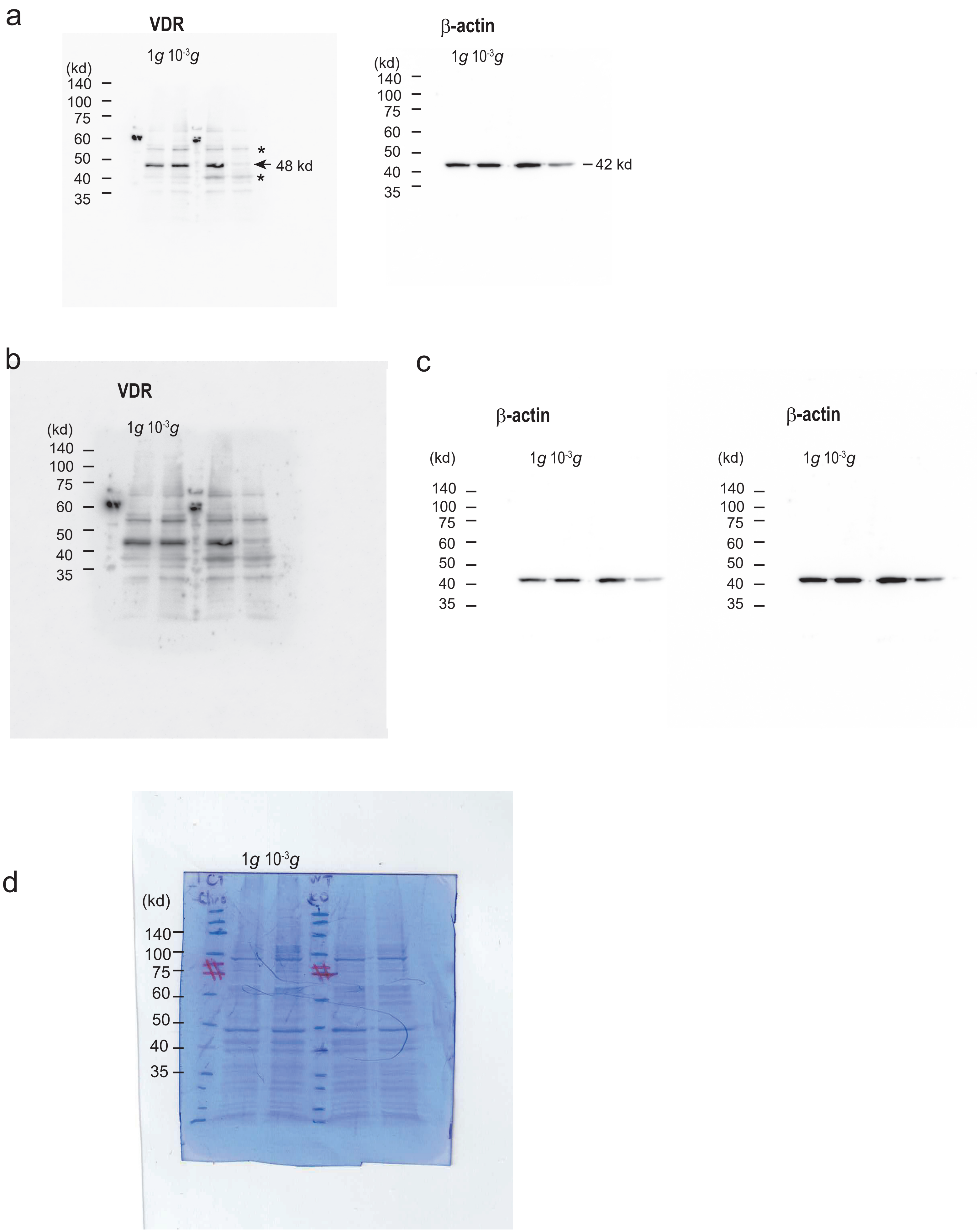
a
VDR
1g 10-3g
(kd)
140
100
75
60
50
40
35
48 kd
β-actin
42 kd
b
VDR
c
β-actin
d

Suppl. Fig.1

a

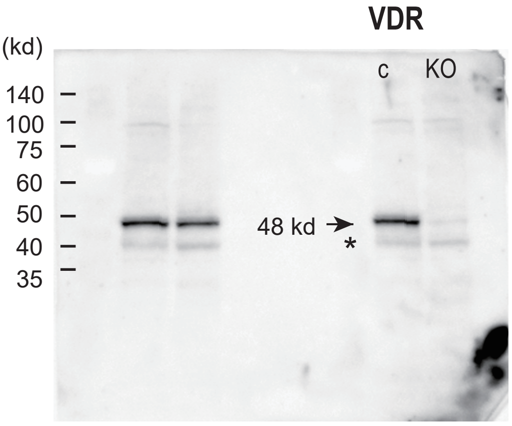

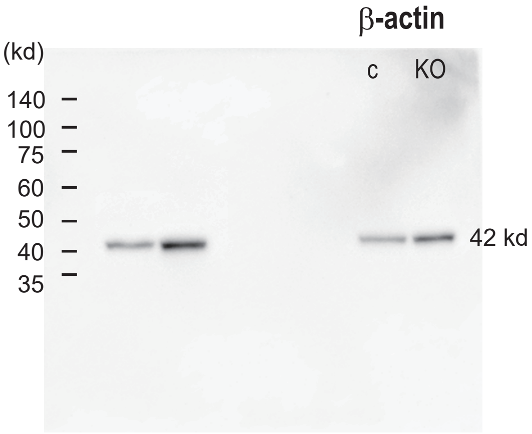

b

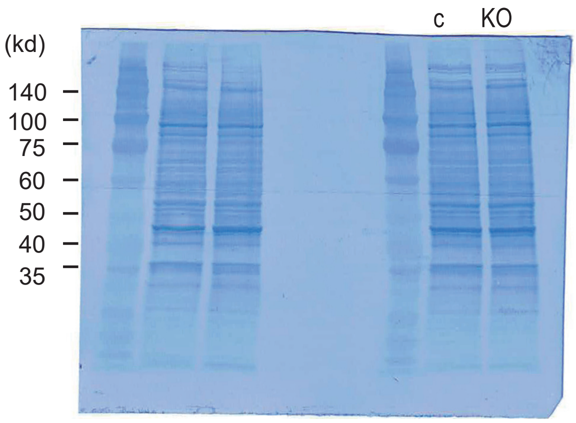

Suppl. Fig.2

a

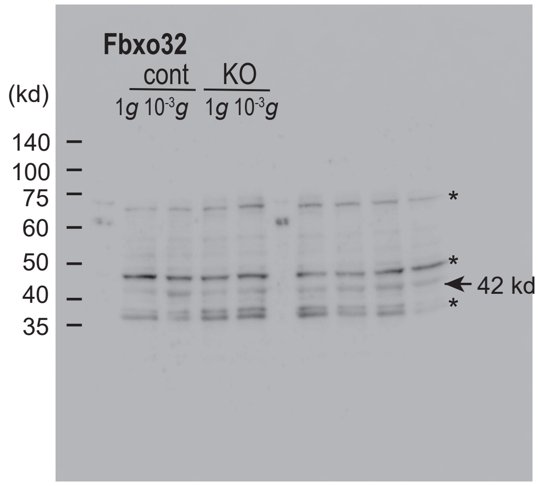

b

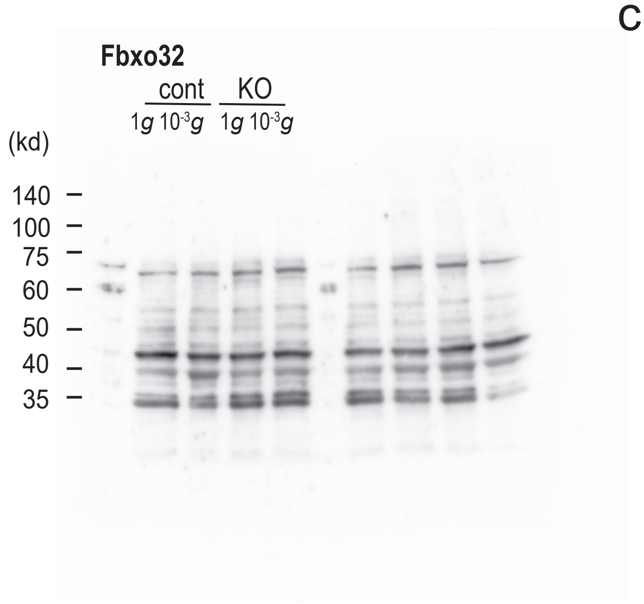

c

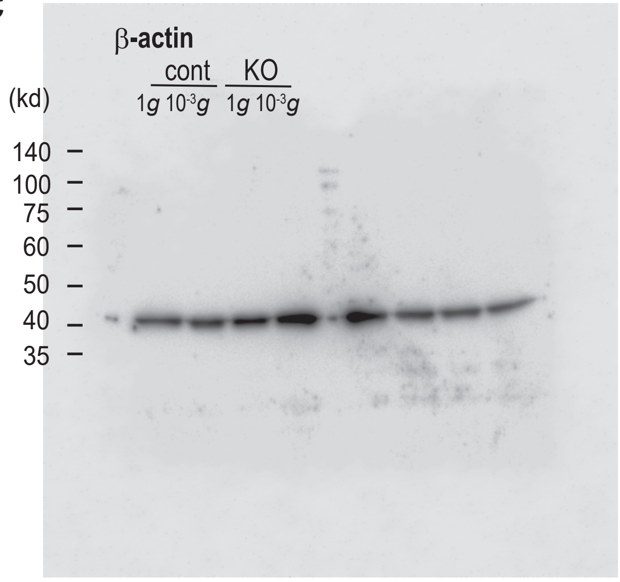

d

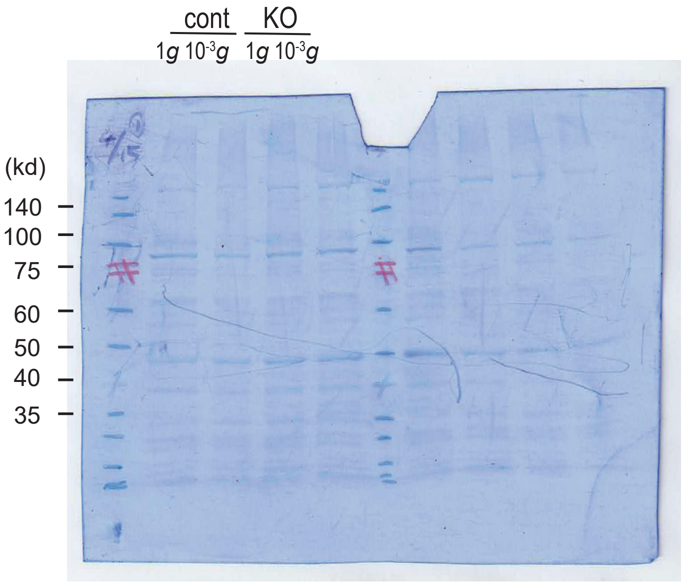

Suppl. Fig.3

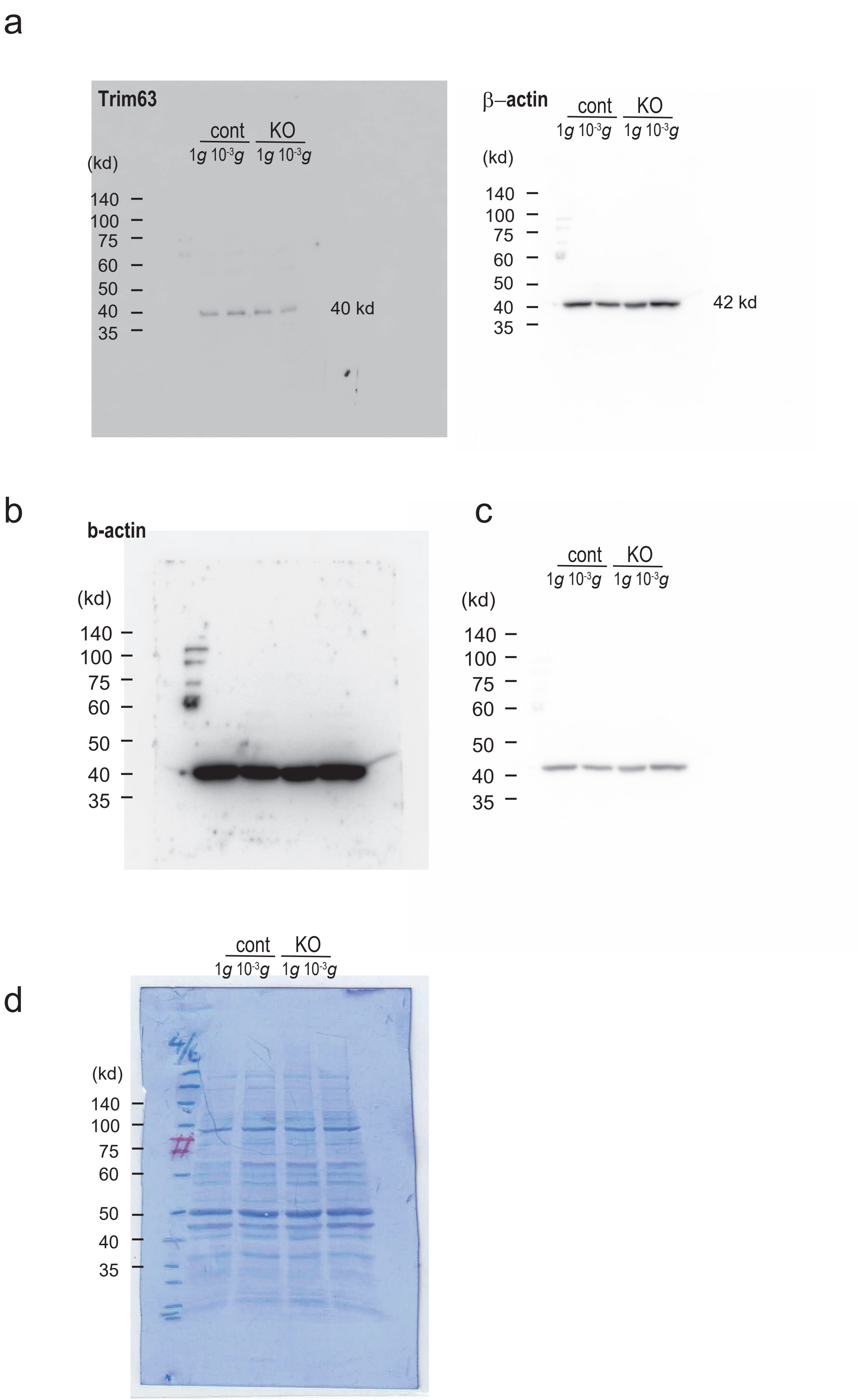
a
Trim63
cont KO
1g 10-3g 1g 10-3g
(kd)
140
100
75
60
50
40
35
40 kd
β–actin
cont KO
1g 10-3g 1g 10-3g
(kd)
140
100
75
60
50
40
35
42 kd
b
b-actin
(kd)
140
100
75
60
50
40
35
c
cont KO
1g 10-3g 1g 10-3g
(kd)
140
100
75
60
50
40
35
d
cont KO
1g 10-3g 1g 10-3g
(kd)
140
100
75
60
50
40
35

Suppl. Fig.4

Figure legends for supplemental figures

Supplemental Figure 1

a) Images of the original blots corresponding to Figure 2b. The membrane was trimmed prior to hybridization with the antibodies. The membrane hybridized with VDR antibody was reprobed with β-actin antibody. The arrow shows bands corresponding to VDR.

b) An original image of VDR with a longer exposure time.

c) Original images of β–actin with different exposure times.

d) CBB staining of the membrane. Protein Ladder One Plus Triple-color for SDS-PAGE (Nacalai Tesque) was used as the molecular marker.

Supplemental Figure 2

a) Images of the original blots corresponding to Figure 3b. The membrane hybridized with VDR antibody was reprobed with β-actin antibody. The arrow shows bands corresponding to VDR. *: non-specific bands.

b) CBB staining of the membrane. Protein Ladder One Plus Triple-color for SDS-PAGE (Nacalai Tesque) was used as a molecular marker.

Supplemental Figure 3

a) Images of the original blots correspond to Figure 4d. The membrane was trimmed prior to hybridization with antibodies. The arrow shows bands corresponding to Fbxo32.

*: non-specific bands.

b) An original image of Fbxo32 with a longer exposure time.

c) An image of β-actin with a longer exposure time. The membrane hybridized with Fbxo32 antibody was reprobed with β-actin antibody.

d) CBB staining of the membrane probed for Fbxo32 and β-actin. Protein Ladder One Plus, Triple-color for SDS-PAGE (Nacalai Tesque) was used as a molecular marker.

Supplemental Figure 4

a) Images of the original blots correspond to Figure 4d. The membrane was trimmed prior to hybridization with antibodies. The membrane hybridized with Trim63 antibody was reprobed with β -actin antibody.

b) An original image of β-actin with a longer exposure time.

c) An original image of β–actin with a shorter exposure time.

d) CBB staining of the membrane probed for Trim63 and β-actin. Protein Ladder One

Plus Triple-color for SDS-PAGE (Nacalai Tesque) was used as a molecular marker.
